# Study on the cytokines related to SARS-Cov-2 in testicular cells and the interaction network between cells based on scRNA-seq data

**DOI:** 10.1515/biol-2022-0661

**Published:** 2023-08-08

**Authors:** Fan Yu, Haihong He, Tingting Huang, Yiwen Zhou

**Affiliations:** Center of Clinical Laboratory Medicine, Shenzhen Hospital, Southern Medical University, Shenzhen, Guangdong, 518000, China; Center of Clinical Laboratory Medicine, Shenzhen Hospital, Southern Medical University, No. 1333 Xinhu Road, Shenzhen, Guangdong, 518000, China

**Keywords:** cytokine, SARS-CoV-2, COVID-19, testicular cell, single-cell RNA-Seq

## Abstract

Inflammatory cytokine storms (CS) in COVID-19 patients are associated with elevated levels of 13 specific cytokines, potentially impacting male fertility by causing testicular cell damage and disrupting the immune microenvironment. Some patients present with scrotal discomfort and orchitis. However, few studies have explored cytokine expression in testicular cells and their role in cell-to-cell communication. In this study, we integrated single-cell sequencing data sets of testicular cells, annotating 20 cell clusters using marker genes and the Human Cell Landscape database. We constructed cell pseudo-chronological trajectories, hub genes, and analyzed the cytokine interaction network between sperm cells using CellChat. Our findings identified 12 types of testicular cells, with four cytokines (IL8, CCL2, CCL3, and TNF) potentially involved in immune processes. Pseudo-chronological trajectory analysis indicated IL8 and CCL3's essential roles in testicular macrophages and endothelial cell development, affecting the immune microenvironment. We determined eight key cytokines (IL1, IL2, IL4, IL6, CCL, CSF3, TNF, and IFN-II) functions in cell interaction networks. Network analysis of exogenous cytokines directly acting on testicular cells showed IL2 potentially affecting all testicular cells, suggesting a vital role in cell communication. This research offers valuable insights into CSs effects on testicular cells and their potential impact on male fertility during COVID-19 infection.

## 1 Introduction

Despite extensive vaccination campaigns, public health measures, and medical advancements, the COVID-19 pandemic continues to pose a substantial threat to global health in 2023 [1]. According to the World Health Organization COVID-19 dashboard (https://covid19.who.int/), as of January 2, 2023, there have been over 740 million confirmed cases of COVID-19 worldwide, with more than 6.7 million deaths. The angiotensin-converting enzyme 2 (ACE2) receptor plays a crucial role in COVID-19 pathogenesis: cells expressing high levels of ACE2 may be targeted by SARS-CoV-2, leading to various symptoms in the body [2]. Research has discovered that ACE2 expression in the testis primarily occurs in vas deferens cells, spermatogonia, mesenchymal cells, and supporting cells [3,4,5,6]. These findings suggest that the testis could be a potential target for SARS-CoV-2.

The spike (S) protein of SARS-CoV-2 binds to the ACE2 receptor and interacts with the cellular serine protease (TMPRSS2), initiating S protein activation [7]. Consequently, the virus may enter sperm cells and disrupt the sperm formation process, potentially negatively affecting male fertility and raising the possibility of sexual transmission. Furthermore, a previous pathological biopsy study revealed that testicular inflammation, extensive germ cell destruction, and sperm damage might be complications of SARS-CoV [6]. A recent case report described epididymitis in a 14-year-old boy as a complication of COVID-19 [8]. Hallak's study also reported scrotal discomfort and orchitis symptoms in some COVID-19 patients, but insufficient evidence exists regarding SARS-CoV-2 presence in semen [9]. While numerous studies have not detected the virus in the semen of COVID-19-infected patients [10], testicular biopsy of COVID-19-positive cadavers shows inflammatory cell infiltration [11,12]. Consequently, in COVID-19 patients, the inflammatory cytokine storm (CS) could be a crucial mechanism causing male testicular cell damage.

CS is an uncontrolled, dysfunctional immune response involving continuous activation and expansion of lymphocytes, monocytes, and macrophages. This process secretes numerous cytokines, potentially leading to systemic inflammation, multiple organ failure, and acute respiratory distress syndrome [13]. CS is prevalent in COVID-19 patients [14]. Research indicates that 13 cytokines, including IL1, IL2, IL4, IL6, IL7, IL8, IL10, CSF3, IP-10, CCL2, CCL3, TNF-α, and IFN-γ (INF-Ⅱ), are elevated in COVID-19 patients [15,16,17,18,19,20]. Proper testicular function and male reproductive system regulation depend on normal cytokine activity [21]. Therefore, CS-induced testicular cell damage and immune microenvironment disruption in COVID-19 patients might affect male fertility. However, few studies have examined the expression of different cytokines in testicular cells and their role in cell-to-cell communication.

In this study, we aimed to identify cytokines that influence testicular cell development and the interaction network between testicular cells based on bioinformatics analysis. Our goal was to explore the potential mechanisms of cytokine signaling pathways that affect male fertility in COVID-19 patients.

## 2 Materials and methods

### 2.1 Data sources

The ten single-cell sequencing data sets used in this study were obtained from the GEO database (http://www.ncbi.nlm.nih.gov/geo), including six data sets from GSE109037 and four data sets from GSE124263. The six data sets from GSE109037 were labeled spermatids1, spermatids17, spermatocytes1, spermatocytes17, spermatogenesis, and spermatogonia. The four data sets from GSE124263 were single-cell sequencing from two adult testicular cells, either enriched for ITGA6 expression or unfractionated before GEM capture. By merging the two data sets, we increased the sample size and enriched the research scope, thereby improving the accuracy of our analysis results.

### 2.2 Single-cell sequencing data analysis and cell cluster annotation

We integrated the data sets from GSE109037 and GSE124263 using the Seurat package (version 3.2.2) [22] in R software (version 4.02). Batch effect removal and normalization of the data sets were performed using Harmony [23]. Principal component analysis and non-linear dimensionality reduction based on UMAP were applied for cell clustering. The FindAllMarkers function was employed to obtain highly variable marker genes of cell clusters, and the heatmap of cell cluster correlations was visualized using the pheatmap package (version 1.0.12). Cell clusters were annotated in combination with the Human Cell Landscape database (http://bis.zju.edu.cn/HCL/).

### 2.3 Construction of single-cell and hub gene trajectories

Pseudotime for all germ cells was generated using Monocle 2 [24], based on the highly variable genes identified by Seurat. Data dimensions were reduced using the discriminative dimensionality reduction with trees (DDRTree) method. All states were generated in pseudo-chronological order, and the pseudotime track of the cytokine was drawn. The plot_genes_in_pseudotime function was employed for visualization.

### 2.4 Analysis of cytokine interaction network between sperm cells using CellChat

The CellChat package [25], which combines social network analysis, pattern recognition, and multiple learning methods, was utilized to quantitatively describe and compare inferred cell–cell interaction networks. The netVisual_aggregate function was used to visualize the communication network of the signaling pathway, while the netVisual_individual function was applied to visualize the communication network of the single ligand–receptor pair (L–R pair) related to the signaling pathway.

## 3 Results

### 3.1 Integrated analysis of single-cell sequencing data sets

**Figure 1:**
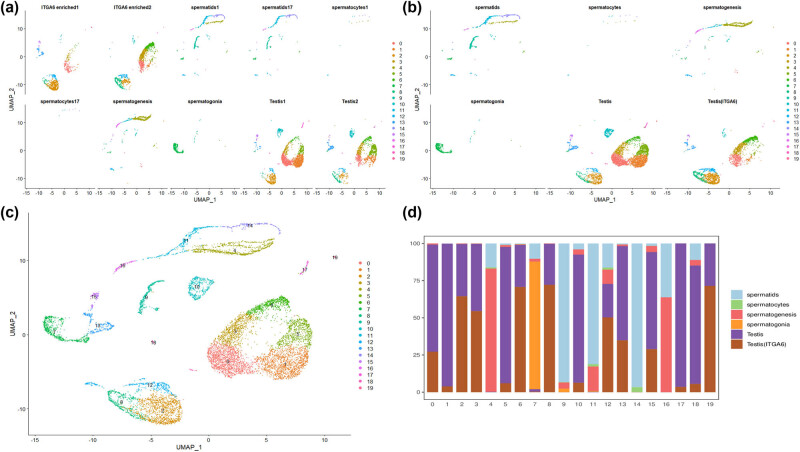
Single-cell sequencing data integrated by Seurat: (a) distribution of 10 data sets, (b) 6 cell groups merged from the data sets, (c) an integrated data set containing 20 cell clusters, and (d) the stacked graph showing the proportion of six cell groups in the cell clusters.

The six data sets from GSE109037 and the four data sets from GSE124263 were integrated for analysis. The GSE109037 data set comprises 2,722 cells, while the GSE123263 data set contains 7,963 cells. The cell count for the ten datasets is displayed in Figure 1a. Subsequently, these datasets were classified into six groups based on cell source (Figure 1b). The final data set consisted of 10,663 cells, 32,360 genes, and 20 cell clusters (Figure 1c). The cell count for each of the six groups is as follows: 5,052 cells in the Testis group, 2,889 cells in the Testis (ITGA6) group, 1,275 cells in the spermatids group, 878 cells in the spermatogenesis group, 543 cells in the spermatogonia group, and 26 cells in the spermatocytes group. The proportions of these cells in the cell clusters are illustrated in Figure 1d.

### 3.2 Annotation of testicular cell clusters and analysis of cytokine expression in cell clusters

**Figure 2:**
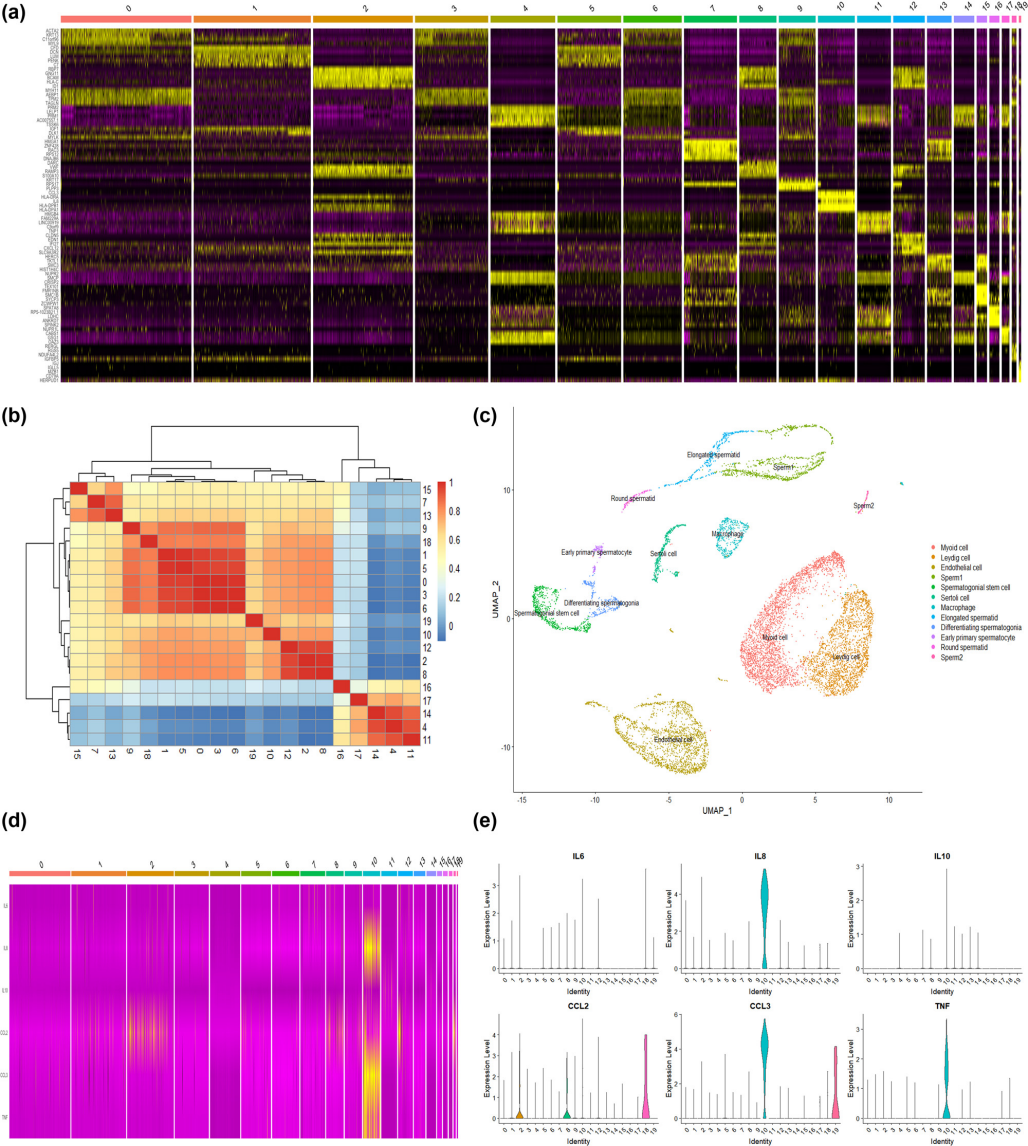
Identification of testicular cell clusters and cytokine expression: (a) heat map of cell clustering marker genes, (b) heat map of the correlation of each cell cluster, (c) the distribution of 12 cell clusters, (d) the heat map of the six cytokines selected in the integrated data set, and (e) the distribution of 6 cytokines in each cell cluster.

The FindAllMarkers function was utilized to identify and visualize the top five marker genes for each cell cluster (Figure 2a). Based on the cell cluster correlation analysis (Figure 2b) and the HPL database, we identified 12 types of sperm cells within the 20 cell clusters (Figure 2c). Cell clusters 7, 13, and 15 were classified as spermatogonial stem cells, differentiating spermatogonia, and early primary spermatocyte cells, respectively, all of which were in the meiotic stage of sperm cells. Cell clusters 4 (14), 11, 16, and 17 were identified as sperm1, elongated spermatid, round spermatid, and sperm2 cells, respectively, which were in the forming stages of sperm cells. Cell clusters 2, 8, 12, and 18 were endothelial cells, while clusters 10 and 19 were macrophages. Out of the 13 cytokines, only six cytokines (IL6, IL8, IL10, CCL2, CCL3, and TNF) were expressed in testicular cells (Figure 2d). Only four cytokines (IL8, CCL2, CCL3, and TNF) were highly expressed in macrophages and endothelial cells, while they exhibited low or no expression in other testicular cells (Figure 2e).

### 3.3 Construction of trajectories of testicular cells and analysis of the role of cytokines in the development of testicular cells

**Figure 3:**
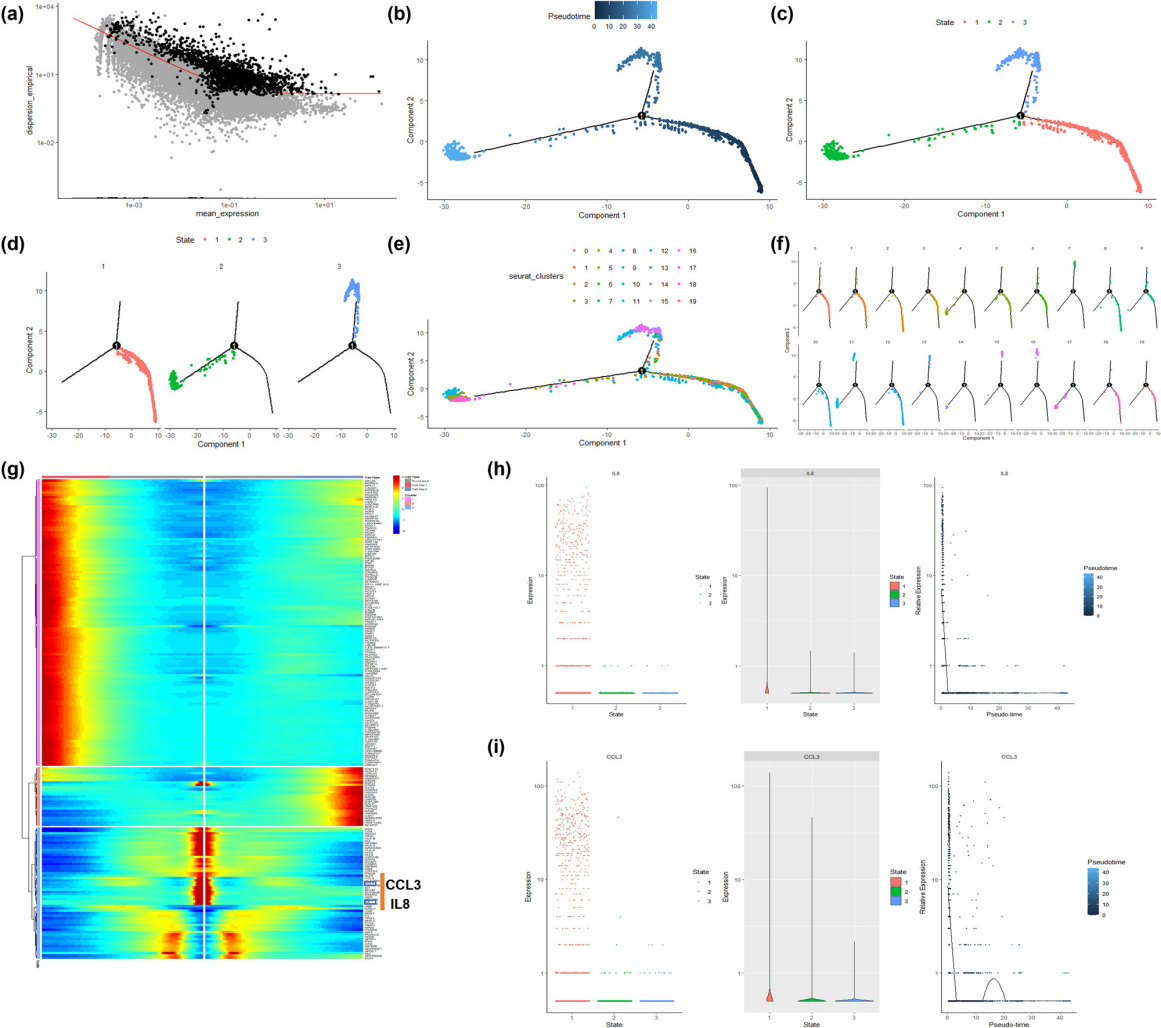
Pseudotime time analysis of testicular cells: (a) Scatter plot of gene expression levels. The black dots are genes that are abnormally mutated between cells for cluster analysis. (b) The pseudo-chronological analysis of testicular cells shows that a branch appears during the development of testicular cells. (c and d) The results of pseudo-time sequence analysis show that there are three states in total. (e and f) The specific positions of 20 cell clusters in state, among which Macrophage and Endothelial cells are mainly enriched in state 1. (g) Heat map of the differential genes among the three state cells. The CCL4 and IL8 genes show a trend from high expression to low expression in state 1. (h and i) The expression of CCL4 and IL8 genes in the developmental trajectory of testicular cells.

The developmental trajectory of sperm cells revealed a branch in the pseudotime analysis of testicular development (Figure 3a and b), resulting in the division into three states (Figure 3c and d). Among these states, macrophages and endothelial cells were mainly enriched in state 1 (Figure 3e and f). According to the heatmap results, among the 13 cytokines, only IL8 and CCL3 exhibited a significant decreasing trend in cells transitioning from state 1 to state 2 (Figure 3g–i). This suggests that IL8 and CCL3 might play crucial roles in testicular macrophage and endothelial cells. Based on these effects, IL8 and CCL3 might further influence the immune microenvironment of testicular cells.

### 3.4 Identification of key cytokines and analysis of their interaction network between testicular cells

**Figure 4:**
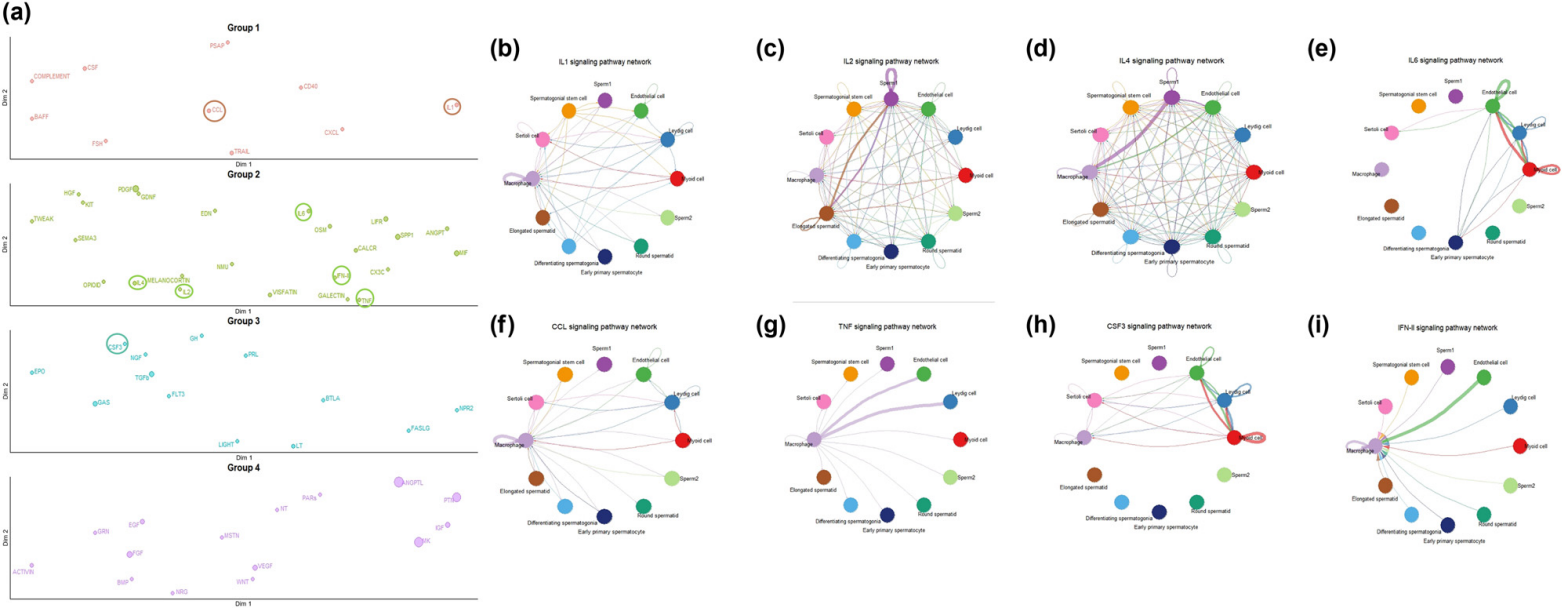
Key cytokines that play a central role in testicular cells. (a) According to function classification, eight key cytokines out of 13 cytokines play a role in testicular cells (circled part). (b) IL1 single pathway network of testicular cells, (c) IL2 single pathway network of testicular cells, (d) IL4 single pathway network of testicular cells, (e) IL6 single pathway network of testicular cells, (f) CCL single pathway network of testicular cells, (g) TNF single pathway network of testicular cells, (h) CSF3 single pathway network of testicular cells, and (i) IFN-Ⅱ single pathway network of testicular cells.

Testicular cells were classified according to their functions using the CellChat package, and the results revealed a total of 62 pathways (4 groups) acting in testicular cells. Among the 13 inflammatory cytokines, eight cytokines directly affected testicular cells by participating in these pathways. They were mainly distributed in three groups: IL1 and CCL in groups 1, IL2, IL4, IL6, IFN-II (IFN-γ), and TNF in group 2, and CSF3 in group 3 (Figure 4a). In all testicular cells, IL1, IL2, IL4, and CCL-related signaling pathways formed a complex interaction network. IL1 had a stronger role in non-spermatogenic cells than in spermatogenic cells (Figure 4b). The IL2 signaling pathway exhibited a strong interaction between elongated spermatid and sperm1 cells (Figure 4c). The IL4 signaling pathway had a strong effect on macrophages, endothelial cells, and sperm1 cells (Figure 4d). CCL mainly affected the interaction between macrophages and other testicular cells (Figure 4f). The IL6 signaling pathway network primarily acted in non-spermatogenic cells (Figure 4e). Both TNF and IFN-II signaling pathway networks were related to macrophages (Figure 4g and i). The CSF3 signaling pathway network interacted only between non-spermatogenic cells (Figure 4h).

### 3.5 Identifying imported cytokines that can affect all testicular cells

**Figure 5:**
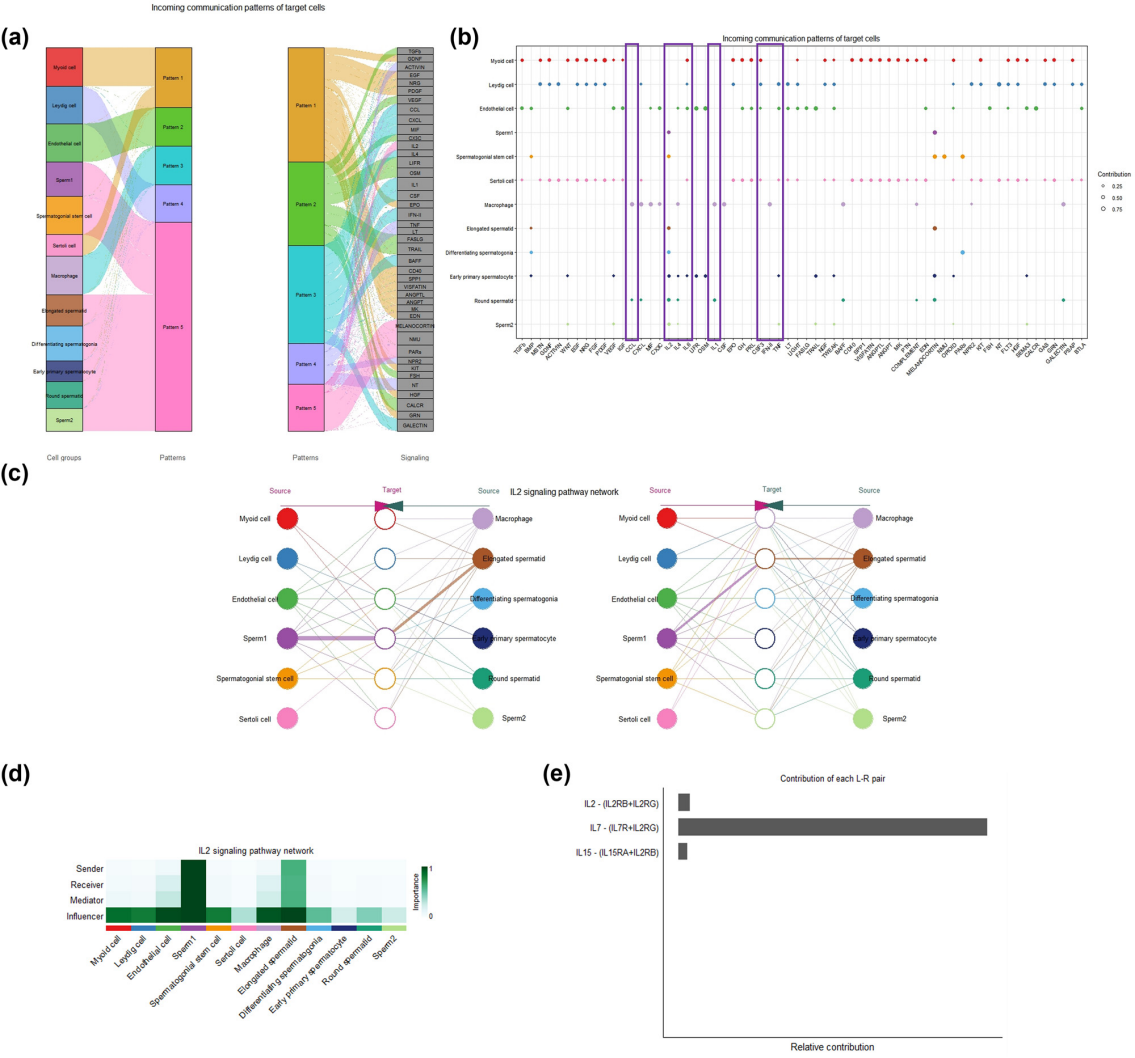
Recognition of cytokines that can affect all testicular cells: (a) The mulberry chart showing that all testicular cells are divided into five patterns according to the functional classification, and IL2 directly acts on the cells of pattern 5. (b) The bubble chart showing the testicular cells that eight key cytokines directly affect. The imported IL2 can directly affect all testicular sperm cells. (c) The network diagram showing that the IL2 single pathway between testicular cells can affect all testicular cells. (d) The heat map showing that the elongated spermatid and sperm1 participate in the sender, receiver, and mediator of the IL2 signaling pathway. (e) The bar plot showing the relative contribution of the ligand-receptor pairs for cell interaction.

Testicular cells were divided into five patterns based on their functions. Sperm cells were all collected in pattern 5, and the imported cytokine IL2 directly affected pattern 5 cells (Figure 5a). Among the eight key cytokines, only IL2 affected all sperm cells (Figure 5b), and signaling pathway analysis showed that it affected all testicular cells (Figure 5c). This indicated that IL2 produced by patients with CS could directly impact all sperm cells and affect other testicular cells, leading to extensive damage to spermatogenesis. Among the cells, elongated spermatid and sperm1 contained the sender, receiver, and mediator of IL2 cell signaling, while all testicular cells expressed the influencer of IL2 cell signaling (Figure 5d). The ligand–receptor pairs for cell interaction included IL2-(IL2RB+IL2RG), IL7-(IL7R+IL2RG), and IL15-(IL15RA+IL2RB) (Figure 5e).

## 4 Discussion

The testicular immune privilege protects immunogenic germ cells from the host's immune response. Viruses, including hepatitis B virus, human immunodeficiency virus, human herpes virus, Ebola virus, and Zika virus, can disrupt this balance and have even been detected in human semen or testicular tissues, leading to orchitis [26,27]. These viruses can adversely affect male reproductive function and sperm production, potentially resulting in fetal malformations [28]. Furthermore, the correlation between elevated cytokine levels in patients with COVID-19 infection and those caused by mumps orchitis has been noted [29,30,31].

Nowadays, by integrating and analyzing large-scale omics data, bioinformatics methods are instrumental in revealing potential molecular mechanisms underlying diseases, including cancers and inflammatory diseases [32,33]. A recent review study identified 13 elevated cytokines (IL1, IL2, IL4, IL6, IL7, IL8, IL10, CSF3, IP-10, CCL2, CCL3, TNF-α, and IFN-γ) in patients infected with COVID-19 [20]. In our study, by integrating single-cell sequencing data of testicular cells, 20 cell clusters were formed, and 12 types of testicular cells were classified. IL8, CCL2, CCL3, and TNF were found to be highly expressed in macrophages and endothelial cells but scarcely present in other testicular cells. This finding suggests that under normal circumstances, these four factors might play a role in testicular immunity. A previous study has identified CCL2 as a functional testicular immune biomarker, demonstrating a significant positive correlation between CCL2 levels and testicular immune infiltration levels [34]. In another investigation focused on the immunology of neonatal prepuce tissue, it was revealed that an elevated expression of TLR4, along with its associated cytokines and chemokines such as TNF-α, CCL2, and CCL3, could potentially enhance innate immunity and contribute to combating pathogens [35]. Pseudo-chronological analysis of testicular cells revealed that IL8 and CCL3 play crucial roles in the development of testicular macrophages and endothelial cells. This finding aligns with Guazzone's study [36], which reported that CCL3 plays a significant role in recruiting immune cells to the testis, with the peak CCL3 content in the testis coinciding with the disease onset.

Eight key cytokines (IL1, IL2, IL4, IL6, CSF3, CCL, TNF, and IFN-II) were identified in the interaction between testicular cells, exhibiting significantly different interactions. Among them, the network involving IL1, IL2, IL4, and CCL signaling pathways was the most complex, potentially affecting all testicular cells. A recent review highlighted that numerous varicocele-related studies involving both animal models and human patients, consistently reported an abnormal increase in the levels of pro-inflammatory cytokines, including IL1 and TNF-α, within seminal plasma, testicular tissue, and peripheral blood. These observations emphasize the involvement of these cytokines in activating potential inflammatory pathways in testicular cells [37]. In another study focusing on chromium-induced testicular toxicity in male rats, the pro-inflammatory roles of IL1, IL6, IL10, and TNF-α in testicular cells were also mentioned [38]. The IL6 signaling pathway network mainly affected non-spermatogenic cells, with early primary spermatocytes being the primary targets among spermatogenic cells. The TNF and IFN-II signaling pathways networks revealed that all testicular cells influenced macrophages. Previous studies have also demonstrated that elevated levels of IL1, IL6, CCL, TNF, and IFN-II in semen are associated with decreased sperm count, sperm motility, and sperm morphology [39,40,41]. Current research indicates that the 13 cytokines may be elevated in COVID-19 patients, resulting in a CS that affects organs throughout the body. Based on our analysis, only IL2 among these 13 cytokines can directly or indirectly affect all testicular cells. Researchers have observed significantly higher IL2 levels in the testes of LPS sepsis rats compared to the normal control group [42]. Another study has also suggested that the reduction of IL1 and IL2 levels by lycopene may contribute to the improvement of hypoxia-induced testicular injury [43]. Although the etiology of the CS varies, these findings are consistent with our research. Integrating our findings with corroborating literature, we propose that IL2 plays a pivotal role in testicular damage during the CS induced by COVID-19 infection. However, the specific role of IL2 and other binding relationships between the ligand and receptor still require further experimental validation. This represents a limitation of the current study.

## 5 Conclusions

COVID-19 patients are prone to developing a CS. Based on existing literature, it has been found that 13 cytokines may increase. By integrating multiple single-cell sequencing datasets, we identified 12 types of testicular cells and discovered that four of them might play a role in the immune process of testicular cells. Through constructing a pseudo-chronological analysis of testicular cells, we determined that IL8 and CCL3 may play crucial roles in the development of testicular immune cells, consequently affecting the immune microenvironment of testicular cells. We further identified eight key cytokines, each playing a significant and distinct role in the network between testicular cells. After analyzing the network of exogenous cytokines directly acting on testicular cells, we concluded that IL2 plays a vital role in testicular cell communication.
